# Cholangiocarcinoma protective factors in Greater Mekong Subregion: Critical issues for joint planning to sustainably solve regional public health problems

**DOI:** 10.1371/journal.pone.0262589

**Published:** 2022-01-27

**Authors:** Nopparat Songserm, Somkiattiyos Woradet, Waratip Kankarn, Kanjanar Pintakham, Phouthong Vanhnivongkham, Nguyen Thi To Uyen, Nguyen Cong Cuu, Le Ngoc Cua, Banchob Sripa, Akhtar Ali

**Affiliations:** 1 Faculty of Public Health, Ubon Ratchathani Rajabhat University, Ubon Ratchathani, Thailand; 2 Faculty of Health and Sports Science, Thaksin University, Phatthalung, Thailand; 3 Faculty of Nursing, Ubon Ratchathani Rajabhat University, Ubon Ratchathani, Thailand; 4 Faculty of Health Sciences, Chiang Rai Rajabhat University, Chiang Rai, Thailand; 5 Champasak Health Science College, Champasak, Lao PDR; 6 Dong Thap Medical College, Dong Thap, Vietnam; 7 Faculty of Allied Health Sciences, Mekong University, Vinh Long, Vietnam; 8 Faculty of Medicine, Khon Kaen University, Khon Kaen, Thailand; 9 WHO Collaborating Centre for Research and Control of Opisthorchiasis, Tropical Disease Research Laboratory, Faculty of Medicine, Khon Kaen University, Khon Kaen, Thailand; 10 Department of Biological Science, The University of Tulsa, Tulsa, Oklahoma, United States of America; George Washington University, UNITED STATES

## Abstract

Although *Opisthorchis viverrini* (OV), lifestyle, and diet co-factors have a relatively high prevalence in the Greater Mekong Subregion (GMS) population, cumulative (0–74) incidence rates of cholangiocarcinoma (CCA) do not reach 5% in this region. Other co-factors must influence, but in this study, we only highlighted positive factors for guiding joint planning to address public health problems at the regional level. Therefore, we aimed to study prevalence and factors associated with CCA incidence focusing only on protective factors. A cross-sectional analytic study was carried out from June to October 2017. Participants with informed consent completed the questionnaires. Descriptive statistics were used to analyze general information. Primary variables were classified into high and low levels by mean. Logistic regression was employed to investigate the correlation between interesting variables and the overall risk level of CCA. The overall prevalence of CCA protective factors of the whole region was knowledge (61.39%), health beliefs (42.32%), prevention behavior (31.93%), and community participation (14.53%). When considering the proportions at a high level, they were 49.53%, 53.72%, 35.37%, and 49.67%, respectively. Significant factors associated with CCA prevention were females with secondary or vocational education, a high level of perceived seriousness and benefits, and community participation. These findings are likely to be helpful for both the public and administrators. First, it can be information for people to be aware of CCA risk. Second, policy-driven authorities at the local or regional level should apply the critical issues from this study for joint planning to sustainably solve regional public health problems.

## Introduction

The Greater Mekong Subregion (GMS) are lowlands with many tributaries that flow into the Mekong River. The Mekong River runs through six countries, including China, Myanmar, Laos, Thailand, Cambodia, and Vietnam, and it is recorded as the top ten longest rivers in the world [1]. It is undeniable that this river has formed people’s culture, beliefs, religion, and way of life. Hence, people have similar traditions, beliefs/religions, ways of life, and food consumption culture. But, unfortunately, the people living in this region are not only the resource-poor countries in Asia [2], but they also have a high risk of *Opisthorchis viverrini* (OV) infection and cholangiocarcinoma (CCA) [3]. Worse still, although the incidence of both diseases is high in rural residents, especially those with poor social status [4], they still do not get attention in the western world. Therefore, they are classified as neglected tropical diseases [5].

If the focus is on studying the risk factors of CCA, OV infection is a significant risk factor [6–8]. In addition, lifestyle and diet co-factors, such as eating raw fish and nitrosamine-containing foods [9–11] and alcohol drinking [12], are also the risk factors found in subsequent studies. On the contrary, the CCA protective factor is the consumption of fruits and vegetables [10]. Later, the study results were confirmed employing meta-analysis used to determine what factors were statistically related to the occurrence of CCA. The main findings revealed the following five risk factors: alcohol, OV, praziquantel, raw fish, nitrosamines [13], and two protective factors, namely vegetables and fruits [13, 14]. Shockingly, people in some areas in the GMS have a very high prevalence of OV, such as more than 70% in Thai people [3] and 85% in Lao people [15]. In addition, the population in five GMS countries has a relatively high percentage of exposure to these five risk factors (50.2%; range 2.0–77.2) [16]. China was excluded from such study [16] because significant liver fluke transmission is *Clonorchis sinensis* (CS), not OV, like the other five GMS countries. Although the above risk factors have a relatively high prevalence in the GMS population, the cumulative (0–74) incidence rates of CCA do not reach 5% in this region. Thus, for example, only the cities with a cancer registry are mentioned: Thailand (Khon Kaen Province) and Vietnam (Ho Chi Minh City) [17]. Therefore, it is inevitable that there are still other co-factors that influence the incidence of CCA. However, in this research, we highlighted only the positive factors for guiding joint planning to address public health problems at the regional level.

So far, there has been no empirical evidence to support decision-making on CCA management and resource allocation and planning for preventing CCA in each country. Furthermore, another observation revealed that the individual factors that may influence CCA incidence in this region had not been collected, especially in knowledge, health beliefs, prevention behavior, and community participation in CCA prevention. Therefore, this research aimed to study the prevalence and the factors associated with CCA incidence among rural people along the Mekong River in the GMS, focusing only on the protective factors. The findings are expected to be beneficial for two levels: (1) the public: It will be the information for people to be aware of the risk of CCA to find ways to prevent and reduce the risk. (2) The executives or those with authority in driving the policy: It will be the information for CCA prevention and risk reduction planning in each country as well as a guideline for joint planning for sustainably solving regional public health problems.

## Materials and methods

### Study design

A cross-sectional analytic study was employed to study this region from north to south (Myanmar to Vietnam) from June to October 2017. The details of the research conduction have been presented in our previous research [16]. Briefly, trained researchers and research assistants of each country collected data in the areas using the questionnaire created by the research team.

### Study setting

**Fig 1:**
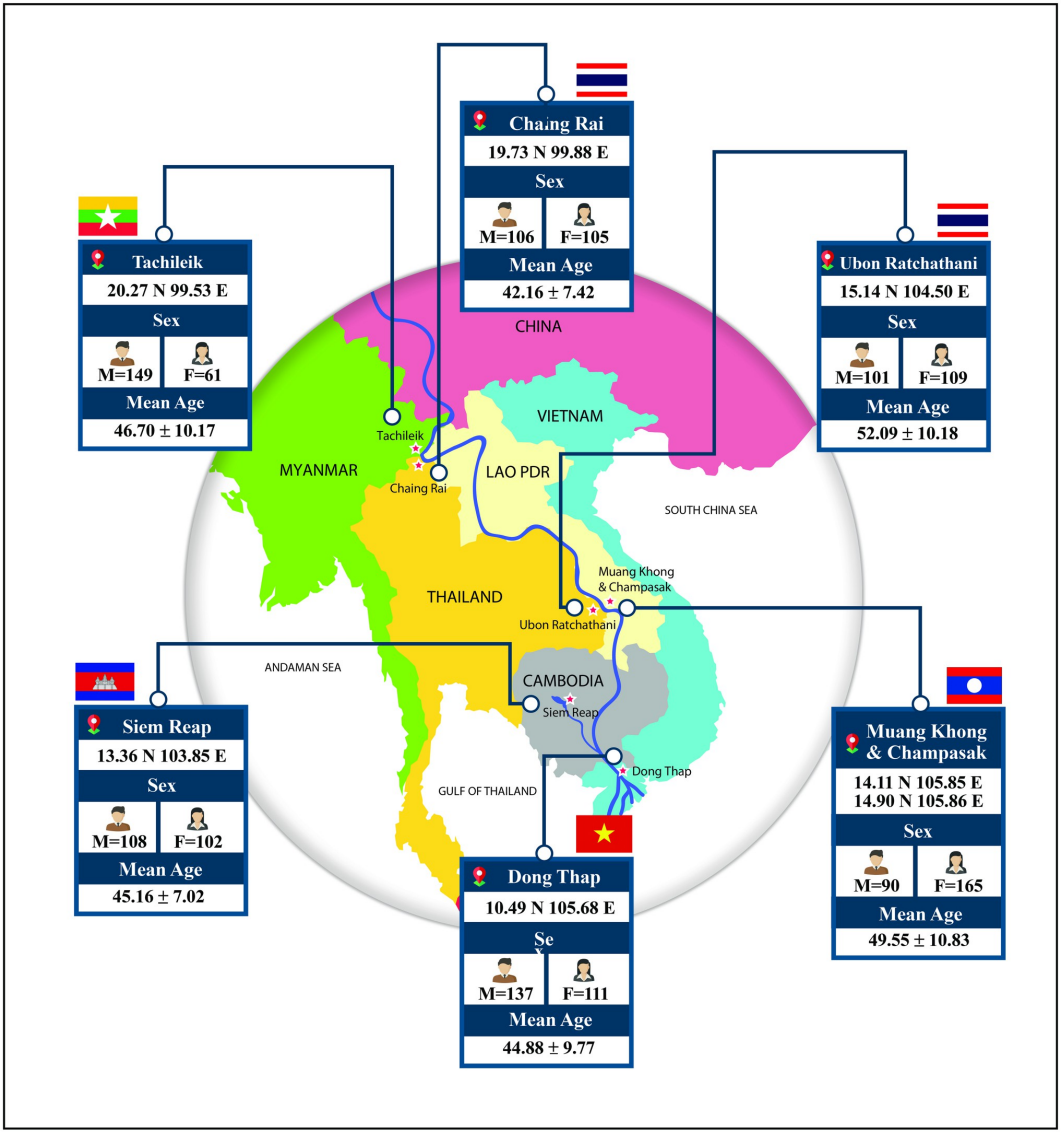
The study areas classified the general information of study participants.

The study area consisted of six settings in five GMS countries. The details of the designation of the study setting were previously described [16]. In this study, the data were presented based on geography, as explained in Fig 1. Tachileik, Myanmar (the abbreviation for this study: TK, MYA), located at 20.27 N 99.53 E.; Chiang Rai, Thailand (CR, THA), located at 19.73 N 99.88 E.; Ubon Ratchathani, Thailand (UB, THA), located at 15.14 N 104.50 E.; Muang Khong and Champasak, Lao PDR (CP, LAO), located at 14.11 N 105.85 E, 14.90 N 105.86 E, respectively; Siem Reap, Cambodia (SR, CAM), located at 13.36 N 103.85 E. and Dong Thap, Vietnam (DT, VIE), located at 10.49 N 105.68 E.

### Ethical approved

The Ubon Ratchathani Rajabhat University Ethics Committee for Human Research approved this research under the Declaration of Helsinki and the Declaration of Helsinki and the ICH-GCP Guidelines (Reference Number HE601010). The ethical considerations adhered to respect for a person, risk and benefit, justice, potential risks, and protection of volunteers’ information. The researchers explained every step of the project to the volunteers. When the volunteers decided to join the project, they were asked to sign a consent form. The researchers respected the decision and provided advice on health issues to the volunteers.

### Study participants

Based on our research’s previous published paper [16], the study participants were people living in the rural areas of five GMS countries (Myanmar, Laos, Thailand, Cambodia, and Vietnam). We calculated the sample size based on the sample size calculation formula. When substituting values in the procedure, 1,251 samples were obtained. Still, since we conducted this research in six research areas, the proportion of the sample was equally divided, that is, 210 people/area. So, the total number was 1,260 samples. The process of sampling and the total number of complete questionnaires are shown in Table 1. The inclusion criteria were people between 30–69 years who voluntarily participated in the project. The exclusion criteria were as follows: those who did not wish to provide information and could not participate in the entire research process. The general statement of the samples classified by the study areas is presented in Fig 1.

**Table 1:** Process of sampling and number of complete questionnaires.

Research areas	The total number of questionnaires collection	The number of refused to respond or incomplete data (%)	The total number of complete questionnaires	Percentage per region
TK, MYA	220	10 (4.54)	210	15.63
CR, THA	220	9 (4.09)	211	15.69
UB, THA	220	10 (4.54)	210	15.63
CP, LAO	270	15 (5.55)	255	18.97
SR, CAM	220	10 (4.54)	210	15.63
DT, VIE	270	22 (8.15)	248	18.45

### Research tools and quality test

The data collection tool (questionnaire) was constructed based on the data from related research. The content was scoped before creating a questionnaire, and the scoring criteria were set for each answer. The questionnaire consisted of 6 parts. Part 1 was general information, consisting of the closed-ended questions used to inquire about socioeconomic status. Part 2 was CCA risk factors, consisting of the questions with answer choices about the history of exposure and the frequency of exposure to risk factors. The details of the questions in the first two parts have already been presented [16, 18]. Part 3 was CCA knowledge. Part 4 was the health beliefs of CCA. Part 5 was CCA prevention behavior. Part 6 was community participation in CCA prevention. The questionnaire was translated into the local language and validated by five experts, three university lecturers specializing in health education and behavioral sciences, and two healthcare workers specializing in disease prevention and control. The reliability of the questionnaire was tested by trying out with another group of 30 samples and analyzed for Cronbach’s Alpha Coefficient (α). Overall, the reliability of each item was more significant than 0.80.

### Data collection

Data collection consists of two stages. (1) The preparatory stage consisted of preparing people involved in the study (researchers, research assistants, and volunteers), places, research tools, and equipment for field visits, and contact with community leaders in that area. (2) The operational stage consisted of informing the volunteers of the purposes and procedures of data collection, obtaining informed consent from the volunteers, conducting data collection, verifying completeness of data, and conducting statistical analysis.

### Outcome measures

Ten questions assessed the CCA knowledge. The questions consisted of two response choices (yes, and no). A score of "1" was for the correct answer, and "0" was for the incorrect answer.

The health beliefs of CCA were assessed based on four key areas: (1) perceived susceptibility (5 items), (2) perceived seriousness (5 items), (3) perceived benefits (5 items), and (4) perceived barriers (5 items). Each question consisted on a 3-point scale: “1” = disagree, “2” = not sure, and “3” = agree.

CCA prevention behavior was assessed by 10 questions with 3-point scale: "1" = never, "2" = sometimes, and "3" = regularly.

Community participation in CCA prevention was assessed by 10 questions with a 3-point scale: "1" = never, "2" = sometimes, and "3" = regularly.

### Statistical analysis

All data were analyzed by a statistical package, SPSS version 26.0 (IBM Company, Chicago, USA). A p-value <0.05 was considered statistically significant.

General information, knowledge, health beliefs, prevention behavior, and community participation in CCA prevention were analyzed by descriptive statistics, namely, percentage, mean, and standard deviation.

The levels of primary variables were classified into high and low groups based on mean values. The knowledge levels were cut off at >7 and ≤7 points, respectively. The groups of health beliefs consisted of 4 items, namely perceived susceptibility, perceived seriousness, perceived benefits, and perceived barriers, and the cut-off points were 11.72, 11.93, 12.72, and 12.13, respectively. The prevention behavior levels were cut off at >22 and ≤22 points, respectively. The stories of community participation in CCA prevention were cut at >18 and ≤18 points, respectively. In addition, the overall risk level of CCA was based on previous research [16, 18], which was cut off at >5.53 and ≤5.53 points, respectively.

Comparison of the means of knowledge, health beliefs, prevention behavior, and community participation in CCA prevention was first analyzed using one-way ANOVA. We then used the Bonferroni correction (Bonferroni post hoc test) to investigate the pairs with means of each factor among each pair of the study area.

Univariate and multivariate logistic regression were employed to investigate the correlation between the interesting variables, including gender, age, educational level, knowledge, health beliefs, prevention behavior, community participation in CCA prevention, and the overall risk level of CCA (dependent variable). The obtained data were presented by Odds ratio (OR) and 95% confidence interval (95% CI).

## Results

**Fig 2:**
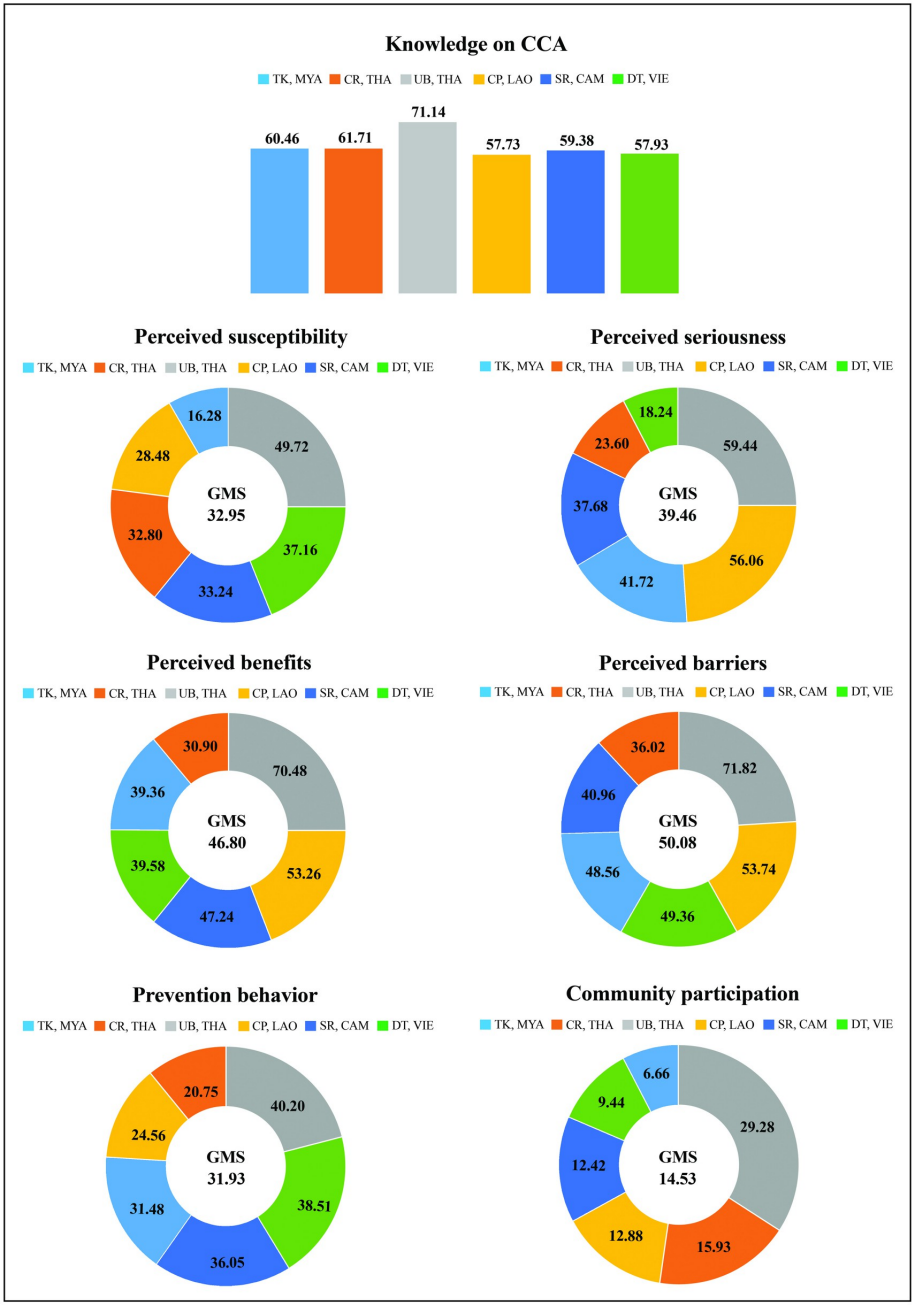
Assessment of cholangiocarcinoma knowledge, health beliefs, prevention behavior, and community participation among rural people residing along the Mekong River in five Greater Mekong Subregion Countries.

Fig 2 presents the assessment results of CCA knowledge health beliefs in 4 items (perceived susceptibility, perceived seriousness, perceived benefits, perceived barriers), prevention behavior, and community participation of rural residents in six areas of five GMS countries.

The results of the assessment of the knowledge about CCA from taking a total of 10 questions, considering only the correct answers, revealed that the average knowledge score of the volunteers in the whole region was 61.39% (max = 71.14% for UB, THA; min = 57.73% for CP, LAO). The question most correctly answered by the volunteers across the region (77.11%) was *"Opisthorchiasis is caused by eating raw or undercooked scaled freshwater fish such as Cyprinid fishes*." On the other hand, the question least correctly answered (37.43%) was *"People who eat raw fish must take a drug to eliminate flukes for at least once a year*".

The mean score of the overall health belief in the region was 42.32%. When assessed separately for each item, the mean score of perceived susceptibility of the whole region was 32.95% (max = 49.72% for UB, THA; min = 16.28% for TK, MYA), and 39.46% of perceived seriousness was the overall outcome. Again, the highest mean score was found in UB, THA, while the lowest was in DT, VIE. Additionally, the mean scores of the whole region’s perceived benefits and perceived barriers were 46.80% and 50.08%, respectively. Interestingly, the highest mean score of these two items was found in UB, THA, while the lowest one was found in CR, THA, respectively.

The mean prevention behavior across the region was 31.93% (max = 40.20% for UB, THA; min = 20.75% for CR, THA). The overall mean score for community participation was 14.53%. The area with the highest mean score was UB, THA, while TK, MYA had the lowest mean score.

**Fig 3:**
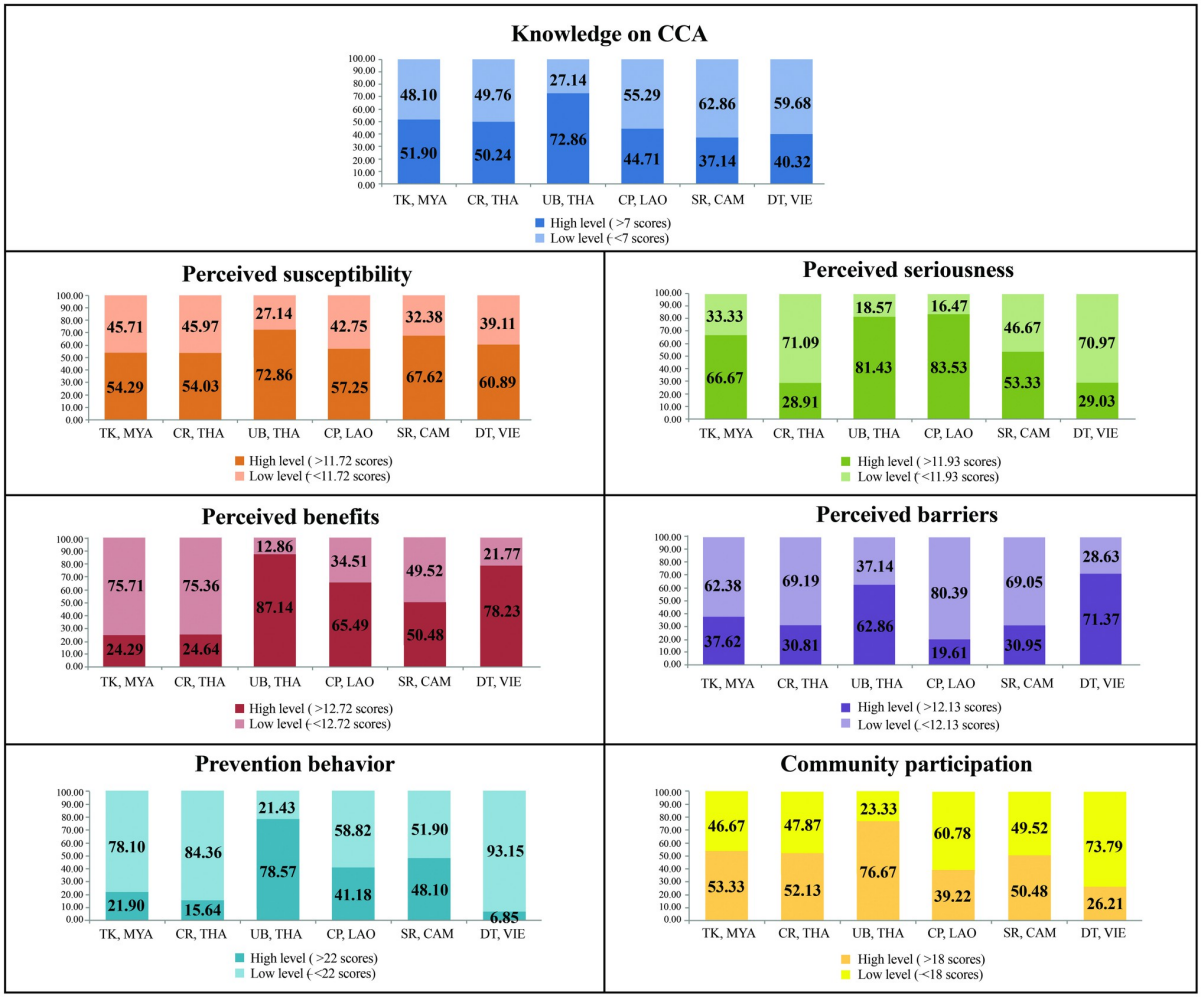
The study areas classified the high and low-risk levels of knowledge, health beliefs, prevention behavior, and community participation in cholangiocarcinoma prevention by mean.

The division of high and low levels of the exciting factors in the study based on mean values was presented in Fig 3. If it took only the knowledge at a high level (>7 scores) into account, UB, THA was found with the most significant proportion (72.86%), while SR, CAM was found with a minor proportion (37.14%). An interesting point in assessing the health belief levels was that the majority of samples of UB, THA had the highest level of perception of the four items. Likewise, evaluating the level of prevention behavior and community participation for CCA prevention revealed that the highest proportion was found in the samples in UB, THA. In contrast, the most negligible proportion was found in the participants in DT, VIE.

Table 2 shows the comparison of the means of knowledge, health beliefs, prevention behavior, and community participation in CCA prevention in each area; they were statistically different (p<0.001 for all factors).

**Table 2:** Comparison of the means of knowledge, health beliefs, prevention behavior, and community participation in cholangiocarcinoma prevention by study areas.

Research areas Factors	TK, MYA	CR, THA	UB, THA	CP, LAO	SR, CAM	DT, VIE	p-value*
	Mean (S.D.)	Mean (S.D.)	Mean (S.D.)	Mean (S.D.)	Mean (S.D.)	Mean (S.D.)	
• Knowledge on CCA	6.05 (1.77)	6.17 (1.43)	7.11 (1.20)	5.77 (1.90)	5.94 (1.39)	5.80 (3.36)	<0.001
• Perceived susceptibility	10.66 (0.98)	10.76 (1.43)	11.60 (2.71)	10.75 (1.53)	11.00 (1.51)	10.82 (0.96)	<0.001
• Perceived seriousness	11.32 (1.67)	9.88 (1.80)	11.80 (1.82)	12.11 (1.33)	10.56 (1.74)	9.99 (2.02)	<0.001
• Perceived benefits	10.42 (1.62)	10.22 (2.10)	12.72 (1.11)	12.11 (1.42)	11.41 (1.43)	11.02 (1.06)	<0.001
• Perceived barriers	11.31 (1.11)	10.82 (1.59)	12.18 (1.93)	10.08 (1.51)	10.76 (1.84)	12.45 (1.62)	<0.001
• Prevention behavior	19.18 (2.84)	18.76 (1.97)	22.38 (2.54)	20.07 (1.95)	20.74 (2.91)	19.27 (1.18)	<0.001
• Community participation	16.06 (4.08)	16.61 (4.78)	18.15 (3.73)	15.02 (2.61)	15.54 (3.87)	12.21 (3.30)	<0.001

* p-value for ANOVA.

**Fig 4:**
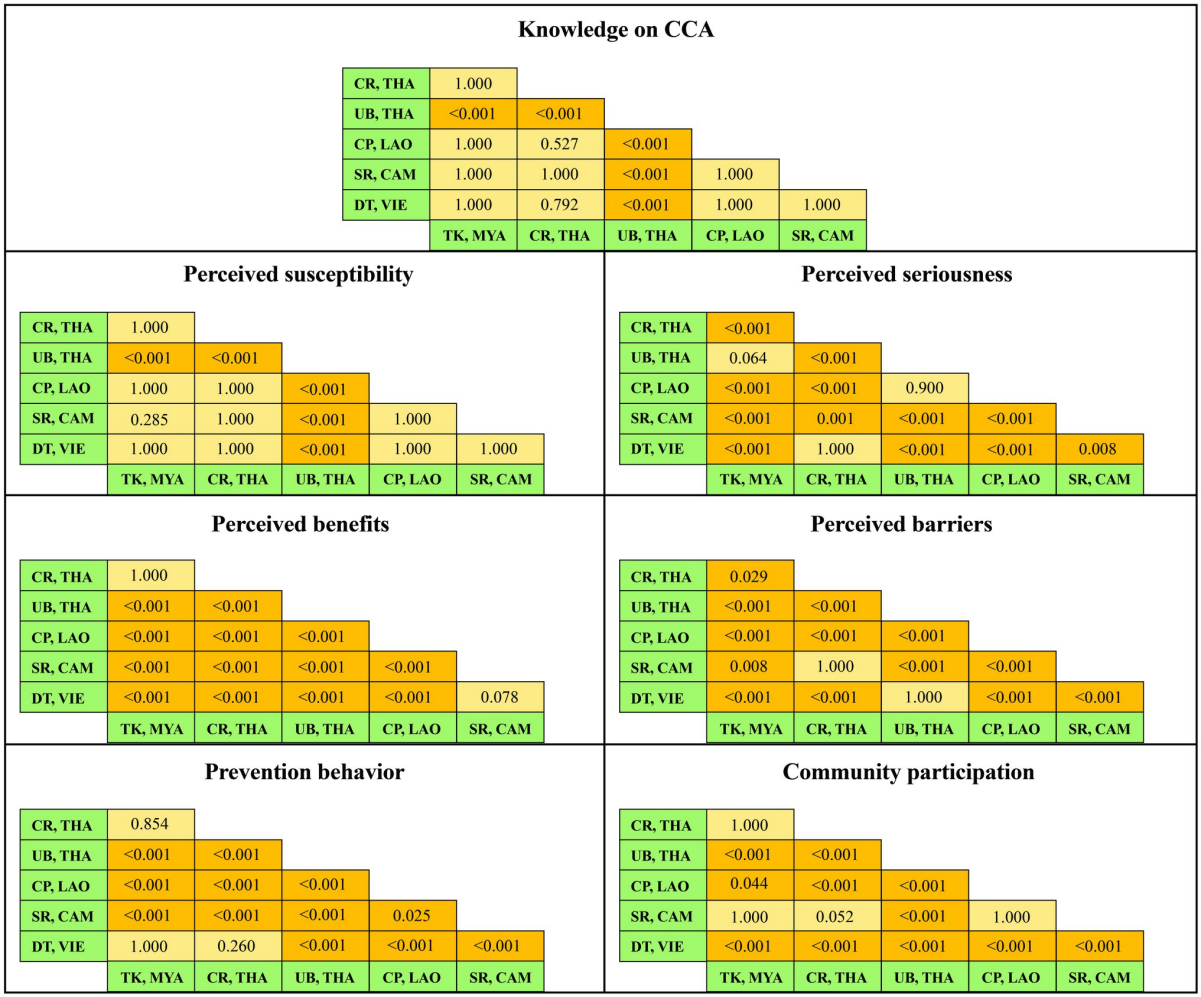
Pair comparisons of the mean scores of knowledge, health beliefs, prevention behavior, and community participation in cholangiocarcinoma prevention by study areas.

When comparing the area in pairs (Fig 4), it was found that the means of knowledge and perceived susceptibility of CCA for UB, THA were statistically different from other areas (p<0.001). Most of the area pairs had a statistically significant difference in perceived seriousness, except for the couples of UB-CP and UB-TK. More importantly, the CR-DT pairings were not different in perceived seriousness and perceived benefits and prevention behaviors. Most of the people in each pair of study areas had a statistically significant difference in community participation, except for SR pairs with other areas (SR-TK, SR-CR, SR-CP). In addition, SR-CR was not different in terms of perceived barriers to preventing CCA.

Table 3 presents the analysis results of the factors associated with the risk of CCA among rural residents of five GMS countries. Females were 0.63 times less risky than males (Adjusted OR, OR_adj_ = 0.63; 95% CI: 0.50–0.79). People with secondary school and vocational education levels were at 0.69 times and 0.66 times less risky than the reference group. People with perceived seriousness and benefits at a high level were at 0.50 and 0.58 times less risky than those with low levels, respectively. On the other hand, people with perceived susceptibility and barriers at a high level were more at risk than the reference group. In addition, people who participated in the community for CCA prevention were less at risk than the other group (OR_adj_ = 0.64; 95% CI: 0.50–0.82).

**Table 3:** Factors associated with the risk of cholangiocarcinoma in the Greater Mekong Subregion.

Factors	Number	%	OR*	OR**	95% CI***	p-value
**Sex**						
• Male	691	51.41	1.00	1.00		
• Female	653	48.59	0.64	0.63	0.50–0.79	<0.001
**Age (years)**						
• ≤44	591	43.97	1.00	1.00		
• 45–59	564	41.96	1.17	1.20	0.93–1.54	0.163
• ≥60	189	14.06	1.35	1.11	0.77–1.60	0.590
**Educational level**						
• Illiterate & Primary school	804	59.82	1.00	1.00		
• Secondary school	281	20.91	0.36	0.69	0.51–0.93	0.016
• Vocational	181	13.47	0.35	0.66	0.46–0.94	0.022
• Diploma or higher	78	5.80	0.52	1.02	0.62–1.68	0.938
**Knowledge on CCA (scores)**						
• Low (≤ 7)	684	50.89	1.00	1.00		
• High (>7)	660	49.11	1.05	1.06	0.83–1.36	0.637
**Perceived susceptibility (scores)**						
• Low (≤11.72)	524	38.99	1.00	1.00		
• High (>11.72)	820	61.01	0.69	1.51	1.17–1.94	0.001
**Perceived seriousness (scores)**						
• Low (≤11.93)	575	42.78	1.00	1.00		
• High (>11.93)	769	57.22	2.20	0.50	0.39–0.64	<0.001
**Perceived benefits (scores)**						
• Low (≤12.72)	731	54.39	1.00	1.00		
• High (>12.72)	613	45.61	1.95	0.58	0.45–0.76	<0.001
**Perceived barriers (scores)**						
• Low (≤12.13)	776	57.74	1.00	1.00		
• High (>12.13)	568	42.26	0.45	2.21	1.73–2.82	<0.001
**Prevention behavior (scores)**						
• Low (≤22)	877	65.25	1.00	1.00		
• High (>22)	467	34.75	1.61	0.97	0.74–1.27	0.811
**Community participation (scores)**						
• Low (≤18)	690	51.34	1.00	1.00		
• High (>18)	654	48.66	1.84	0.64	0.50–0.82	<0.001

*Crude odds ratio.

**Adjusted odds ratio (adjusted for all other variables in the table).

***95% confidence interval for adjusted OR**.

## Discussion

This research aimed to investigate the prevalence and factors associated with CCA incidence, focusing only on protective factors in GMS. According to the study results on the overall prevalence of CCA protective factors of the whole region, the following mean scores were revealed: knowledge (61.39%), health beliefs (42.32%), prevention behavior (31.93%), and community participation (14.53%). However, when considering only the proportion of having all four factors at a high level in the whole region, they were 49.53%, 53.72%, 35.37%, and 49.67%, respectively. Notably, disease prevention behavior was accounted for the lowest proportion. This shows that most people in the region still have bad CCA protection behavior, so some areas were assessed as high-risk, such as UB, THA, and CP, LAO [16]. Therefore, it is not only a challenge for public health workers in carrying out CCA prevention and control in their own country, but it is also the responsibility of public health workers across the region. Therefore, it is necessary to find out a suitable model or method for reducing the risk factors, such as using the model of having village health volunteers in high-risk areas in Thailand with a shortage of health personnel [19] or the parallel program of village health volunteers in the twin cities (Thailand–Laos) [18] to prevent and reduce CCA risk factors. In addition, some high-risk areas have adopted a public health approach [20–23] or one health approach [24, 25] to reduce risk factors or control OV and CCA.

It was also found that the factors associated with CCA prevention in the region with statistical significance were females with secondary or vocational education levels and those with a high level of perceived seriousness and benefits and community participation in CCA prevention. Remarkably, some factors influencing involvement in CCA prevention need to be considered: gender and health behavior [26, 27]. These findings are likely to be useful at two levels for both the public and administrators. (1) It can be the information for people to be aware of CCA risk to find ways to prevent and reduce the risk. (2) It can be the information for CCA prevention and risk reduction planning in each country and a guideline for joint planning for solving regional public health problems.

The risk of people with high levels of perceived susceptibility and perceived barriers was 1.51 times and 2.21 times greater than the reference group, respectively, because the national campaigns for OV and CCA prevention and control have been held for more than 40 years [28–30]. In addition, we also found that the means of knowledge, health beliefs, prevention behavior, and community participation in CCA prevention in each area had statistically different. Especially UB, THA were statistically different from other areas for all factors. It is why there have been campaigns in northeast Thailand to deal with OV and CCA for more than 40 years [28–30] until now [18, 19, 31, 32]. So that people in this area have better knowledge and health beliefs than other areas. Moreover, in the 4.0 range, people can easily access information and technology. As a result, they are well aware of the risks and the barriers to disease prevention. However, risky behavior has not been reduced in any way. This may be due to the familiarity with the same behavior that has been done repeatedly [33]. Consequently, the risk of CCA remains. However, the CCA risk will be reduced if people know the disease severity and perceive the benefits of preventive behavioral modifications. Therefore, policy strategies need to be adjusted as the disease seriousness and the benefits if CCA is prevented.

The limitations of this research are as follows. (1) Weaknesses of study design: a cross-sectional study is a study at an individual level to determine the prevalence of a problem at a time. Researchers must collect data about the factors and the disease at the same time. Consequently, it makes it difficult to conclude if the relationship is the cause of the disease or not. In addition, bias due to inadequate response and misclassification may occur. (2) Limitations of data collection: The study did not include protective factors due to the differences in areas or countries, such as OV and CCA prevention policies or projects.

From the literature review, most researchers have tried to identify and reduce risk factors for preventing CCA. Still, very few have done the opposite: finding out and increasing the protective factors, especially those in individuals. Therefore, this research can be regarded as the starting point of finding out the protective factors to plan for CCA prevention in the future. The public health approach to problem-solving consists of 8 steps as follows: (1) define the problem, (2) identify indicators of the problem, (3) find data for the indicators, (4) identify stakeholders, (5) identify critical determinants, (6) identify intervention strategies, (7) identify implementation strategies, and (8) evaluate [34]. However, applying the public health approach to solving the OV and CCA issues in each area requires consideration of the local context, feasibility, and available resources [31]. In particular, policy-driven authorities at the local or regional level should apply the findings or critical issues from this research for joint planning to sustain regional public health problems.

## Supporting information

S1 Dataset Dataset used during the data analysis. (XLSX)

S1 Questionnaire (PDF)
