# Supplementary material for: Cholangiocarcinoma protective factors in Greater Mekong Subregion: Critical issues for joint planning to sustainably solve regional public health problems
Source: PLoS One. 2022 Jan 27;17(1):e0262589. doi: 10.1371/journal.pone.0262589 (PMC8794208; doi:10.1371/journal.pone.0262589)
Supplement: S1 Questionnaire — (PDF) [file pone.0262589.s002.pdf]

ID

Questionnaire

Health behaviors and risk factors of cholangiocarcinoma: A comparative study in rural areas of the Greater Mekong Subregion Countries

Instructions

1. The questionnaire aims to explore the prevalence of risk factors, knowledge, health beliefs and behaviors in the prevention and control as well as the involvement of the community in the prevention and control of cholangiocarcinoma in the rural areas of the countries in the Greater Mekong Sub-region (Thailand, Laos, Myanmar, Cambodia and Vietnam). It is divided into 6 parts.

   Part 1: General information

   Part 2: Risk factors of cholangiocarcinoma

   Part 3: Knowledge of liver fluke and cholangiocarcinoma

   Part 4: Health beliefs about liver fluke and cholangiocarcinoma

   Part 5: Behaviors in the prevention and control of cholangiocarcinoma

   Part 6: The community participation in the prevention and control of cholangiocarcinoma
2. Please check ✓ in ❑ or complete ☐☐ with accurate information that reflects the reality.
3. Your information will be kept confidential strictly and used only for the study.
4. The names or the information that can be linked to the volunteers will not be appeared in the questionnaire.
5. The data will be analyzed and presented as a whole without the names of the informants.
6. In response to this questionnaire, you will voluntarily answer the questions and at any time and for any reason, you can refuse to answer the questions or stop filling out the questionnaire.

Thank you very much for your kind cooperation.

Researchers

Part 1: General information

1. Gender: ❑ 1 = Male ❑ 2 = Female

2. Age: [______________] years

3. Occupation

❑ 1 = Agriculture ❑ 2 = Trading / doing own business

❑ 3 = Factory employee / private company employee ❑ 4 = Government official/employee / state enterprise employee

❑ 5 = labor / general worker ❑ 6 = Student

❑ 7 = Others, please specify_____________

4. Educational level

❑ 1 = Uneducated ❑ 2 = Primary school

❑ 3 = Junior high school ❑ 4 = High school / vocational certificate

❑ 5 = Diploma or equivalent ❑ 6 = Bachelor's degree and higher

5. The average annual income of all family members living together

❑ 1 = Less than 5,000 Baht (<143 $) ❑ 2 = 5,001-10,000 Baht (143-285 $)

❑ 3 = 10,001-15,000 Baht (285-429 $) ❑ 4 = More than 15,000 Baht (>429 $)

6. Marital status

❑ 1 = Single ❑ 2 = Married / living together

❑ 3 = Widowed ❑ 4 = Divorced / separated

Part 2: Risk factors of cholangiocarcinoma

Instructions: Check ✓ in ❑ or complete ☐☐ with accurate information that reflects the reality.

1. Smoking

1.1 Have you ever smoked? ❑ 1. Yes ❑ 2. No. (Skip to 2)

**Types of cigarettes**	**1 = Smoke 2 = Not smoke**		**Frequency of smoking** 1 = Every day; 2 = Every week; 3 = Every month; 4 = < once a month				**The number of cigarettes Smoking per frequency unit**	**Age started smoking**	**Age that wanted to quit smoking**
	**1**	**2**	**1**	**2**	**3**	**4**			
1.2 Government cigarette with butt	❑	❑	❑	❑	❑	❑	☐☐	☐☐	☐☐
1.3 Government cigarette w/o butt	❑	❑	❑	❑	❑	❑	☐☐	☐☐	☐☐
1.4 Hand-rolled tobacco	❑	❑	❑	❑	❑	❑	☐☐	☐☐	☐☐

1.5 Do you still smoke at the present? ❑ 1. Yes. ❑ 2. No.

2. Beverages containing alcohol

Types of beverages	1 = Drink 2 = Not Drink		Frequency 1 = Every day; 2 = Every week; 3 = Every month; 4 = <once a month				The amount of cc per frequency unit	Age started drinking	Age that wanted to quit drinking
	1	2	1	2	3	4			
Beer	❑	❑	❑	❑	❑	❑	☐☐☐☐	☐☐	☐☐
Wort	❑	❑	❑	❑	❑	❑	☐☐☐☐	☐☐	☐☐
Rice whiskey	❑	❑	❑	❑	❑	❑	☐☐☐☐	☐☐	☐☐
Colored whisky	❑	❑	❑	❑	❑	❑	☐☐☐☐	☐☐	☐☐
Other types of whisky ……...………	❑	❑	❑	❑	❑	❑	☐☐☐☐	☐☐	☐☐

3. History of having liver fluke and the use of the medicine for killing liver fluke (Praziquantel)

3.1 Have you ever checked for liver fluke? ❑ 1. Yes. ❑ 2. No. (Skip to 3.4)

3.2 If yes, do you have liver fluke? ❑ 1. Yes. ❑ 2. No. (Skip to 3.4) ❑ 3. Do not know. (Skip to 3.4)

3.3 If you have liver fluke, do you take the medicine for killing liver fluke (praziquantel)?

❑ 1. Yes. ❑ 2. No. (Skip to 3.4)

3.3.1 If yes, how many times do you take the medicine for **killing** liver fluke (praziquantel)? ☐☐ times

3.3.2 If yes, how many years or months that you have taken the medicine for **killing** liver fluke (praziquantel)?

Specify the number of years: ☐☐ years **or** Specify the number of months: ☐☐ months

3.4 Have you ever taken the medicine for killing liver fluke (praziquantel) in order to **prevent** liver fluke? (You do not have liver fluke or have never checked for it, but you take this medicine.)

❑ 1. Yes. ❑ 2. No.

4. Food consumption (Explanation: During the past year, did you consume the following foods and how often?**)**

Foods	1 = Yes. 2 = No.		Frequency 1 = Every day; 2 = Every week; 3 = Every month; 4 = < once a month			
	1	2	1	2	3	4
4.1 Did you eat <u>raw fish salad</u> (freshwater fish with flake such as carp, Siamese mud carp and ricefishes?	❑	❑	❑	❑	❑	❑
4.2 Did you eat <u>pickled fish</u>?	❑	❑	❑	❑	❑	❑
4.3 Did you eat cooked <u>fermented fish</u>?	❑	❑	❑	❑	❑	❑
4.4 Did you eat <u>soured fish</u>?	❑	❑	❑	❑	❑	❑

Part 3: Knowledge of liver fluke and cholangiocarcinoma

Instructions: Check ✓ in ❑ that you think it is correct.

No.	Knowledge of liver fluke and cholangiocarcinoma (CCA)	Yes	No
1	Liver fluke is caused by eating raw or half-cooked freshwater fish with flake such as Siamese mud carp.	❑	❑
2	Dogs and cats eating raw fish can cause liver fluke.	❑	❑
3	Nitrosamines are chemical compounds in fermented foods and can cause CCA.	❑	❑
4	Water vegetables such as water mimosa, morning glory and spirogyra cause liver fluke and CCA.	❑	❑
5	People with liver fluke are asymptomatic, but liver fluke eggs can be detected in feces.	❑	❑
6	People with liver fluke and CCA often have indigestion, weight loss and jaundice.	❑	❑
7	People who eat raw fish have to take the medicine for killing liver fluke at least once a year.	❑	❑
8	Herbal medicines can cure liver fluke.	❑	❑
9	To prevent liver fluke and CCA is to eat cooked food.	❑	❑
10	Controlling the epidemic of liver fluke can be done by defecation in the toilet.	❑	❑

Part 4: Health beliefs about liver fluke and cholangiocarcinoma

Instructions: Check ✓ in ❑ that reflects the reality.

Topics	Opinions		
	Disagree (1)	Not sure (2)	Agree (3)
**The perception of risk**			
1. Healthy people will have no chance of having liver fluke and cholangiocarcinoma.	❑	❑	❑
2. Having liver fluke and cholangiocarcinoma is the fate of individuals.	❑	❑	❑
3. Everyone who always eats raw fermented fish, raw soured fish and pickled fish will have liver fluke and cholangiocarcinoma.	❑	❑	❑
4 Everyone who always eats raw freshwater fish with flake will have liver fluke and cholangiocarcinoma.	❑	❑	❑
5. Eating raw fish cooked with sour ingredients as lemon juice or red ant will not cause liver fluke.	❑	❑	❑

Topics	Opinions		
	Disagree (1)	Not sure (2)	Agree (3)
**The perception of the severity of disease**			
6. Taking the medicine for killing liver fluke often causes the risk of having cholangiocarcinoma.	❑	❑	❑
7. Liver fluke is not terrible because it can be cured by medicine.	❑	❑	❑
8. Cholangiocarcinoma causes very high cost of treatment.	❑	❑	❑
9. Cholangiocarcinoma afflicts both patients and caregivers.	❑	❑	❑
10. Everyone who has cholangiocarcinoma must die.	❑	❑	❑
**The perception of the benefits of treatment and prevention**			
11. Not eating raw fish results in getting healthy.	❑	❑	❑
12. Those who used to eat raw fish should check for liver fluke eggs.	❑	❑	❑
13. Once liver fluke eggs are detected, people should be quickly cured in order to prevent themselves from cholangiocarcinoma.	❑	❑	❑
14. The medicine for killing liver fluke does not have bad effect on health.	❑	❑	❑
15. Cooking fermented fish before eating is a must since it is the way to destroy carcinogens.	❑	❑	❑
**The perception of the obstacles**			
16. You eat raw fish because you love its taste.	❑	❑	❑
17. Keeping feces to check for liver fluke is disgusting.	❑	❑	❑
18. Checking for liver fluke wastes both time and money.	❑	❑	❑
19. It is necessary to do stool examination before taking the medicine for killing liver fluke even it is cumbersome.	❑	❑	❑
20. You are willing to cook food before eating at all times to prevent liver fluke.	❑	❑	❑

Part 5: Behaviors in the prevention and control of cholangiocarcinoma

Instructions: Check ✓ in ❑ that reflects the reality.

Issues	Practices		
	**Never (1)**	**Sometimes (2)**	**Always (3)**
1. Have you ever brought stool to check for liver fluke eggs?	❑	❑	❑
2. Upon detection of liver fluke eggs, did you take the medicine according to the instructions of health authorities?	❑	❑	❑
3. Have you ever defecated outside the toilet or in the field, such as in the garden or the river?	❑	❑	❑
4. Do you cook freshwater fish with flake before eating?	❑	❑	❑
5. Do you ferment fish for more than six months prior to eating?	❑	❑	❑
6. Do you eat cooked fermented fish?	❑	❑	❑
7. Have you ever eaten soured fish?	❑	❑	❑
8. Have you ever eaten pickled fish?	❑	❑	❑
9. Have you ever eaten raw fish salad/pickled fish/soured fish with alcohol?	❑	❑	❑
10. In the past year, have you ever checked your health such as ultrasound abdomen?	❑	❑	❑

Part 6: The community participation in the prevention and control of cholangiocarcinoma

Instructions: Check ✓ in ❑ that reflects the reality.

Issues	Participation		
	Never (1)	Sometimes (2)	Always (3)
1. Have you ever participated in the activities organized by the community / Health Promoting Hospital / TAO?	❑	❑	❑
2. In each meeting, have you ever been involved in sharing ideas?	❑	❑	❑
3. Have you ever been involved in **searching for the problems of patients with cholangiocarcinoma** with public health authorities?	❑	❑	❑
4. Have you ever been involved in **analyzing the problems of cholangiocarcinoma** with public health authorities?	❑	❑	❑
5. Have you ever been involved in **planning for solving the problems of cholangiocarcinoma** with public health authorities?	❑	❑	❑
6. Have you ever been involved in **doing activities for solving the problems of cholangiocarcinoma** with public health authorities such as providing knowledge via the community broadcast tower or providing knowledge to students?	❑	❑	❑
7. Do you eat cooked freshwater fish to prevent cholangiocarcinoma according to the suggestions of the authorities?	❑	❑	❑
8. Do you cooperate to prevent and control cholangiocarcinoma by defecating in the toilet all the time according to the suggestions of the authorities?	❑	❑	❑
9. Do you cooperate in stool examination to check for live fluke eggs all the time?	❑	❑	❑
10. Do you cooperate in the establishment of the health fund such as paying for liver fluke examination or treatment or prevention and control of liver fluke fund?	❑	❑	❑

☺☺☺☺☺
